# Caregiver Burden and Depressive Symptoms in Stroke Survivors’ Informal Caregivers in a Resource-Constrained Setting: A Cross-Sectional Study From Southern Mexico

**DOI:** 10.7759/cureus.108321

**Published:** 2026-05-05

**Authors:** Marco J Jacinto-Amador, Nallely Rincón Peregrino, Alfredo González-Ayala, Daniela Rincón-Peregrino, Rolando de Jesús Posada-Vasquez, Quitzia L Torres-Salazar

**Affiliations:** 1 Family Medicine, Hospital General de Zona No. 2 con Medicina Familiar, Instituto Mexicano del Seguro Social, Salina Cruz, MEX; 2 Directorate of Teaching, Quality, and Research, Servicios de Salud de Durango, Durango, MEX

**Keywords:** caregiver burden, caregivers, depression, informal care, stroke

## Abstract

Background

Stroke is a leading cause of long-term disability worldwide, with a growing number of survivors requiring assistance with daily activities. In resource-constrained settings, this responsibility is frequently assumed by informal caregivers, potentially leading to caregiver burden and adverse mental health outcomes. However, data from underserved regions remain limited.

Objective

To determine the prevalence of caregiver burden among primary informal caregivers of patients with stroke sequelae and to explore its relationship with sociodemographic factors and depressive symptoms.

Methods

A cross-sectional study with an exploratory analytical component was conducted at a secondary-level hospital of the Mexican Social Security Institute in southern Mexico between January 2024 and June 2025. Primary informal caregivers of patients with stroke sequelae were included using a non-probability consecutive sampling strategy. Caregiver burden was assessed using the Zarit Burden Interview (ZBI), and depressive symptoms were evaluated using the Zung Self-Rating Depression Scale (SDS). Descriptive statistics were used to summarize the data. Comparisons between caregivers with and without burden were performed using the Chi-square test for categorical variables. Continuous variables were compared using the Mann-Whitney U test.

Results

A total of 57 caregivers were included (median age 47 years [q25-q75: 42-58]); 47 (82.5%) were female. The prevalence of caregiver burden was 50 (87.7%), while 25 (43.9%) participants exhibited depressive symptoms. Caregivers with burden were older than those without burden (median 47 vs. 41 years; p = 0.027). A significant association was observed between caregiver burden and relationship to the patient (p = 0.012), with children and spouses more frequently affected. Depressive symptoms were present in 25 (50.0%) caregivers with burden and in 0 (0%) of those without burden (p = 0.013).

Conclusions

Caregiver burden is highly prevalent among informal caregivers of stroke survivors in this resource-limited setting and is closely related to the presence of depressive symptoms. These findings underscore the importance of incorporating caregiver assessment into routine clinical practice and support the need for strategies aimed at improving caregiver support and preparedness, particularly in underserved contexts.

## Introduction

Stroke remains one of the leading causes of long-term disability worldwide and a major contributor to mortality, accounting for a substantial proportion of the global disease burden [1]. It is estimated that up to 87% of stroke-related deaths occur in low- and middle-income countries, highlighting a disproportionate impact on resource-constrained settings. In Latin America, stroke ranks among the leading causes of death and disability, with age-adjusted incidence rates ranging from approximately 73.6 to 108 per 100,000 population in community-based studies, including data from Mexico [2]. Although mortality rates have shown a declining trend [3], improved survival has led to a growing population of stroke survivors living with significant functional impairment, with up to 52% experiencing moderate to severe disability in population-based studies conducted in Latin American settings. This epidemiological shift has important implications not only for patients but also for those who assume their care [2].

As survival after stroke improves, a growing proportion of patients live with persistent neurological deficits requiring assistance in activities of daily living. In many health systems, particularly in low- and middle-income countries, this responsibility is largely transferred to informal caregivers, typically family members, who provide sustained, unpaid care under conditions of limited training and institutional support. This dynamic has effectively shifted part of the disease burden from the patient to the household, creating a parallel and often underrecognized impact on caregiver health [4].

Caregiver burden has been consistently associated with adverse physical and mental health outcomes, including anxiety, depression, and reduced quality of life. Despite this, caregiver well-being is seldom integrated into routine clinical assessment, especially in primary care settings. Existing literature has predominantly focused on high-income contexts, while data from resource-constrained environments (where reliance on informal care is greater) remain limited [5]. In Mexico, and particularly in underserved regions such as the southern Pacific coast, the magnitude and characteristics of caregiver burden in the context of stroke-related disability are insufficiently characterized, limiting the development of targeted support strategies [6].

Understanding the extent of caregiver burden and its relationship with depressive symptoms in these settings is essential to inform more comprehensive models of care. Therefore, this study aimed to determine the prevalence of caregiver burden among primary informal caregivers of patients with stroke sequelae treated in a secondary-level hospital in southern Mexico, and to explore its association with sociodemographic factors and depressive symptoms.

## Materials and methods

A cross-sectional study with an exploratory analytical component was conducted at the General Hospital with Family Medicine No. 2 of the Mexican Social Security Institute (IMSS) in Salina Cruz, Oaxaca, Mexico. The study was carried out between January 2024 and June 2025 in a secondary-level care setting serving an urban and peri-urban population in southern Mexico.

Primary informal caregivers of patients with a confirmed diagnosis of stroke (ischemic or hemorrhagic) and residual neurological sequelae were considered eligible. Caregivers were defined as individuals providing the majority of daily care without financial compensation. Inclusion criteria were: age ≥18 years, active involvement in the care of a patient with stroke sequelae, and provision of written informed consent. Caregivers with cognitive impairment or any condition limiting their ability to complete the assessment instruments were excluded. Participants with incomplete questionnaires or who withdrew consent were eliminated from the final analysis.

The sample size was calculated using the formula for proportions in finite populations, assuming a 95% confidence level, an expected prevalence of caregiver burden of 57% based on prior literature [7], and a precision of 10%. From an estimated population of 112 patients with stroke sequelae, a minimum sample of 52 caregivers was obtained. A non-probability consecutive sampling strategy was used, including eligible caregivers identified through clinical records (ARIMAC system and medical files), starting from the most recent cases and proceeding retrospectively until the required sample size was reached. A total of 57 caregivers were included.

Eligible caregivers were contacted either in person during outpatient visits or by telephone. After informed consent was obtained, participants completed a structured questionnaire including sociodemographic variables (age, sex, educational level, occupation, marital status, area of residence, relationship to the patient, and health insurance status). The primary outcome was caregiver burden, assessed using the Zarit Burden Interview (ZBI), a widely used and validated instrument. Caregiver burden was defined as a total score ≥47 points. Severity was categorized as no burden (≤46), mild to moderate burden (47-55), and severe burden (≥56) [8,9]. 

Depressive symptoms were evaluated using the Zung Self-Rating Depression Scale (SDS), a validated tool for the assessment of depressive symptomatology. For analytical purposes, depressive symptoms were dichotomized as present or absent based on standard cut-off values [10,11]. Both instruments were administered at a single time point during the study period, consistent with the cross-sectional design. They were used exclusively for data collection purposes, and no full reproduction of the questionnaires is included in this manuscript.

Data were recorded in a Microsoft Excel (Redmond, WA, USA) database and analyzed using IBM SPSS Statistics version 26 (IBM Corp., Armonk, NY, USA). Descriptive statistics were used to summarize the study population. Categorical variables were expressed as frequencies and percentages, while continuous variables were described using medians and interquartile ranges (q25-q75), given their non-normal distribution assessed by the Shapiro-Wilk test. For the analytical component, comparisons between caregivers with and without burden were performed using the Chi-square test. A p-value <0.05 was considered statistically significant.

The study was approved by the Local Health Research and Ethics Committee of the IMSS (approval number: R-2024-2001-043). All procedures were conducted in accordance with the principles of the Declaration of Helsinki and national regulations for research involving human subjects. Written informed consent was obtained from all participants prior to data collection. The study was classified as minimal risk, as it involved the administration of questionnaires without intervention or manipulation of biological variables.

## Results

A total of 57 primary informal caregivers of patients with stroke sequelae were included in the analysis. The median age of participants was 47 years (q25-q75: 42-58), and the majority were female (47 [82.5%]). Most caregivers were married (46 [80.7%]) and were predominantly the patient’s children (38 [66.7%]), followed by spouses (16 [28.1%]) (Table 1). 

**Table 1:** Sociodemographic Characteristics of Caregivers and Comparison by Caregiver Burden Status Data are presented as median (q25–q75) for continuous variables and as frequency (n) and percentage (%) for categorical variables. Differences in medians were analyzed using the Mann–Whitney U test, while comparisons between categorical variables were performed using the Chi-square test. A p-value < 0.05 was considered statistically significant.

Variable	Total (N=57)	With burden (n=50)	Without burden (n=7)	p
Age (years)*	47 (42–58)	47 (44 – 58.2)	41 (37-55)	0.027
Sex				
Female	47 (82.5%)	41 (82.0%)	6 (85.7%)	0.644
Male	10 (17.5%)	9 (18.0%)	1 (14.3%)	
Marital status				
Married	46 (80.7%)	42 (84.0%)	4 (57.1%)	0.165
Single	10 (17.5%)	7 (14.0%)	3 (42.9%)	
Widowed	1 (1.8%)	1 (2.0%)	0 (0.0%)	
Relationship				
Child	38 (66.7%)	34 (68.0%)	4 (57.1%)	0.012
Spouse	16 (28.1%)	15 (30.0%)	1 (14.3%)	
Other	3 (5.3%)	1 (2.0%)	2 (28.6%)	

**Figure 1:**
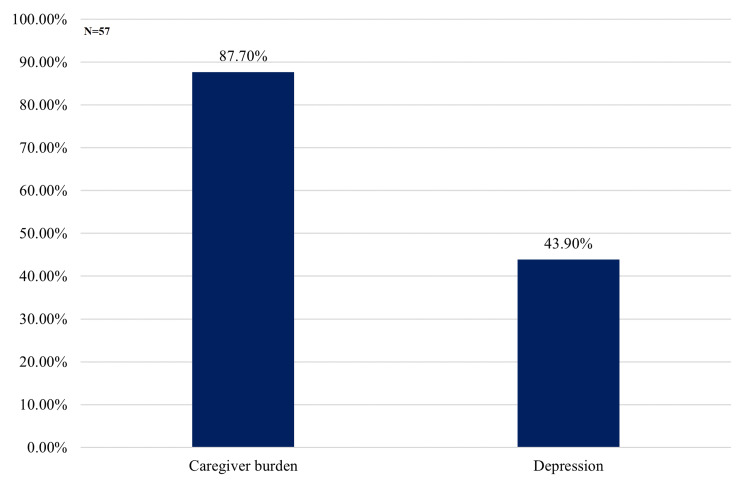
Prevalence of Caregiver Burden and Depression in the Study Population

The prevalence of caregiver burden was high, affecting 50 (87.7%) participants, while 25 (43.9%) exhibited depressive symptoms (Figure 1).

**Figure 2:**
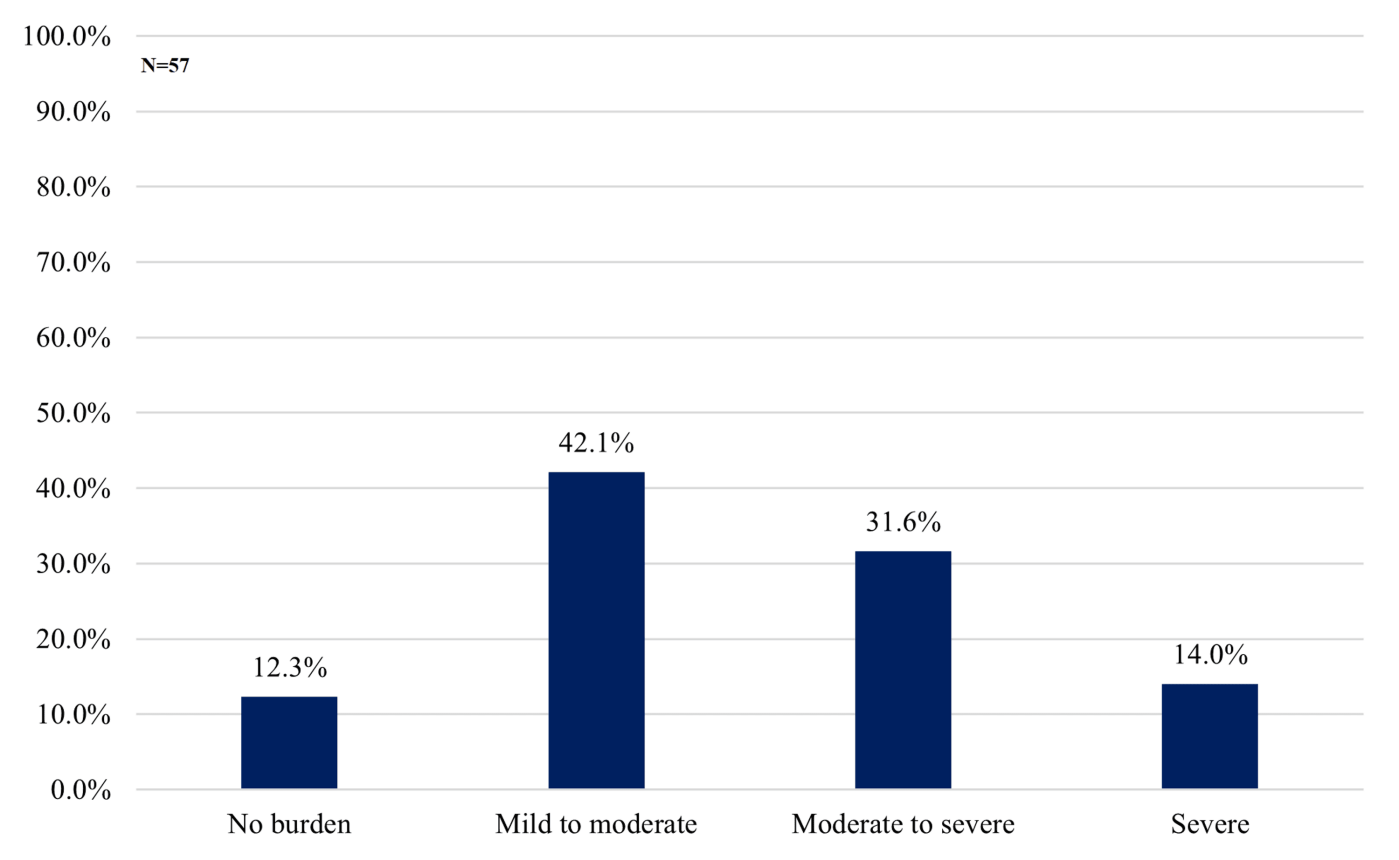
Distribution of Caregiver Burden Severity Based on Zarit Burden Interview Scores

Regarding severity, most caregivers experienced some degree of burden: 24 (42.1%) were classified as mild to moderate, 18 (31.6%) as moderate to severe, and eight (14.0%) as severe burden. Only seven (12.3%) showed no evidence of caregiver burden (Figure 2).

**Figure 3:**
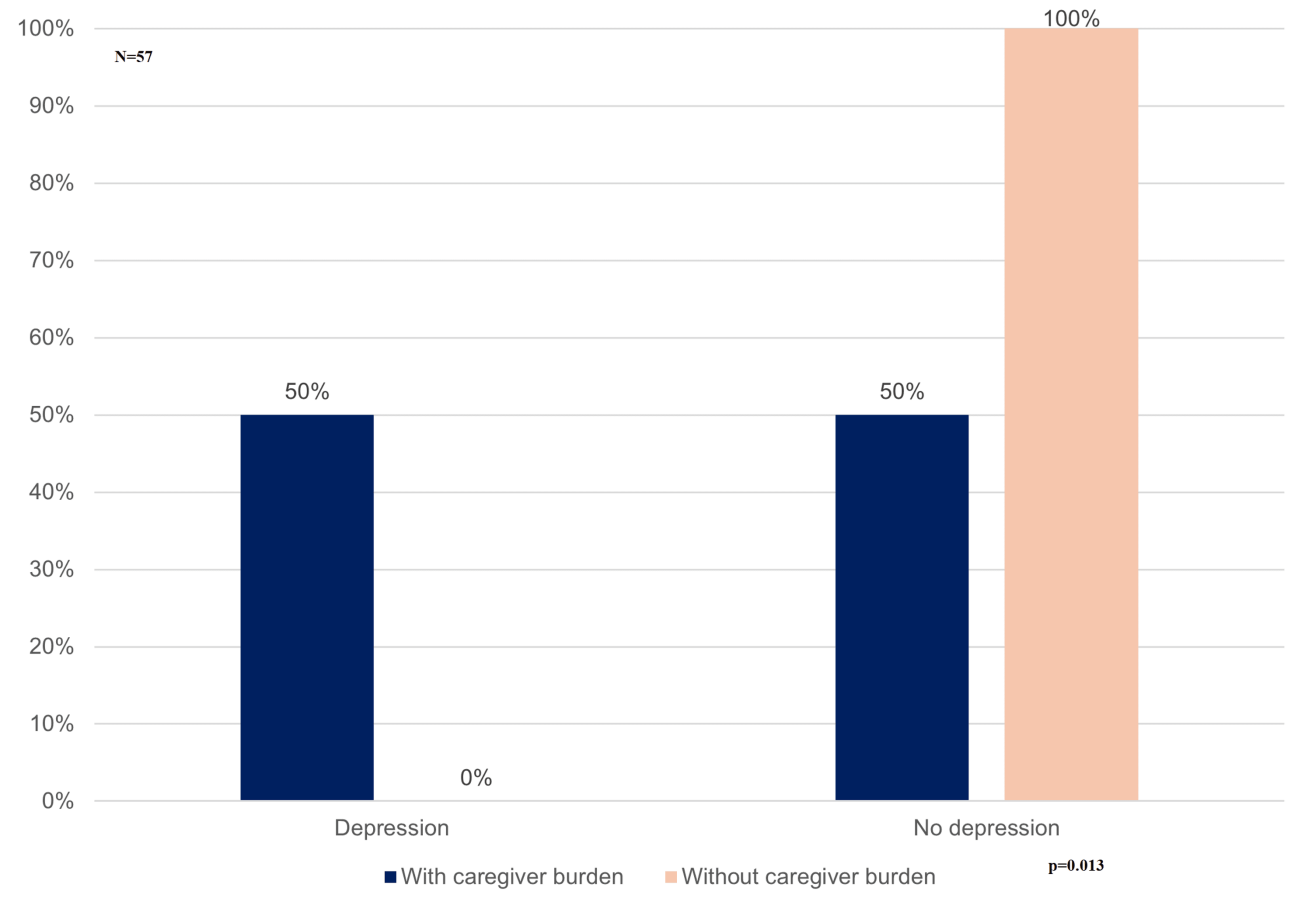
Distribution of Depressive Symptoms According to Caregiver Burden Status Percentages represent within-group proportions. Among caregivers with burden, 50% presented depressive symptoms, whereas none of the caregivers without burden presented depressive symptoms (p = 0.013).

When comparing caregivers with and without burden, a statistically significant difference was observed in age, with those experiencing burden being older (median 47 vs. 41 years; p = 0.027). No significant differences were found for sex (p = 0.644) or marital status (p = 0.165). However, a significant association was identified between caregiver burden and the relationship to the patient (p = 0.012), with children and spouses more frequently affected compared to other relatives (Table 1). Depressive symptoms were more frequent among caregivers with burden: 25 (50.0%) caregivers with burden presented depressive symptoms, whereas none (0%) of those without burden presented depressive symptoms (p = 0.013) (Figure 3).

## Discussion

This study identifies an exceptionally high prevalence of caregiver burden among informal caregivers of patients with stroke sequelae in a resource-limited setting, affecting nearly nine out of 10 participants. This proportion contrasts not only with the more moderate distribution reported by Kazemi et al. [5], in which most caregivers experienced mild to moderate burden, but also with prior literature, including Yaqoob et al. [12], who describe caregiver burden in stroke as frequent and clinically relevant, yet variable across contexts and influenced by available support strategies. These findings highlight the magnitude of an often-overlooked dimension of stroke-related morbidity: the transfer of care demands from formal health systems to households.

One plausible explanation for this elevated prevalence lies in the structural characteristics of healthcare delivery in contexts characterized by heterogeneous access within middle-income countries. Although institutions provide acute care and initial stabilization for patients with stroke, long-term rehabilitation and support services are frequently limited or fragmented. As a result, the continuity of care is largely assumed by informal caregivers, who must compensate for gaps in rehabilitation, follow-up, and social support. This dynamic may shift a substantial proportion of chronic care demands from the health system to families, extending the impact of stroke beyond the individual patient.

Importantly, even in settings with structured rehabilitation programs, caregiver burden has been described as emerging early due to the abrupt assumption of caregiving responsibilities, often accompanied by feelings of inadequacy and lack of preparedness, as reported by Maggio et al. [13]. This suggests that while caregiver burden is an inherent component of post-stroke care, its magnitude may be amplified in contexts where formal support systems are limited, and caregivers must assume complex roles with minimal guidance or institutional backing. This interpretation is consistent with previous literature describing how limited support, restricted access to rehabilitation services, and the complexity of post-stroke care contribute to increased caregiver burden in resource-constrained settings [14].

In this context, caregiver burden should not be interpreted solely as an individual or psychological phenomenon, but also as a potential reflection of broader system-level constraints. The predominance of women, particularly daughters and spouses, among caregivers reflects well-described gender roles in caregiving within Latin American societies, where unpaid care work is disproportionately assigned to female family members. Although Kazemi et al. [5] also reported a predominance of women among caregivers, our findings suggest that in settings with fewer institutional support mechanisms, this distribution may be associated with sustained exposure to caregiving demands, which could contribute to increased perceived burden.

The observed relationship between caregiver burden and depressive symptoms further reinforces the clinical relevance of this issue. In our study, depressive symptoms were only identified among caregivers with burden, suggesting a close relationship between these variables. However, given the cross-sectional design, the directionality of this relationship cannot be determined. This finding is consistent with prior literature indicating that caregiving under conditions of stress and limited support is associated with psychological distress, including depression. For instance, in a large longitudinal cohort, 10.7% of care recipients developed incident depressive symptoms, and those with caregivers experiencing distress, anger, or depression had a 41-45% increased risk of developing these symptoms (adjusted OR ≈1.41) [15], highlighting a clinically relevant interaction between caregiver and patient mental health. Similarly, Uhm et al. reported that caregiver preparedness was strongly and inversely correlated with both caregiver burden (r = −0.512, p < 0.001) and depressive symptoms (r = −0.622, p < 0.001), with more than half of caregivers (55.6%) presenting clinically significant depressive symptoms (CES-D ≥16) [16]. These findings suggest that burden, preparedness, and depressive symptoms are closely interrelated within a broader caregiving context.

Compared with studies from high-income countries, where structured support systems such as respite care, caregiver training programs, and community-based services are more widely available, the caregivers in this study appear to operate within a more constrained support environment. This may contribute not only to the higher prevalence of burden observed, but also to the relatively limited variability in burden severity. In contrast, greater heterogeneity in caregiver experiences has been described in settings where support resources are more accessible. Previous evidence suggests that educational and technology-based interventions may improve caregiver knowledge and patient-related outcomes, although their direct effect on caregiver burden remains variable across contexts [12].

From a public health perspective, these findings support the need to consider stroke care as a dyadic process involving both patients and caregivers. Incorporating caregiver assessment into routine clinical practice, particularly in primary care settings, may facilitate early identification of burden and associated mental health conditions. In addition, interventions such as caregiver education, psychological support, and community-based programs could represent feasible strategies to mitigate the impact of informal caregiving, particularly in underserved settings. Early identification of caregiver burden may therefore represent a critical opportunity to implement targeted interventions aimed at preserving both caregiver well-being and patient outcomes. These findings may also inform the development of structured caregiver support strategies within health systems, including the integration of social support services, caregiver training programs, and early screening protocols in primary care settings.

This study should be interpreted in light of certain considerations. Its cross-sectional design precludes causal inference, and the use of a non-probability sampling strategy may limit generalizability. Although the sample size was calculated using a finite population approach and was adequate for the primary descriptive objective, the analytical component was exploratory, and the imbalance between groups (particularly the small number of caregivers without burden) may have limited the statistical power for subgroup comparisons. Additionally, as this was a single-center study, the findings may not capture the full variability of caregiver experiences across different regions. Further multicenter studies with larger samples are warranted to confirm and extend these findings.

## Conclusions

This study highlights that caregiver burden among informal caregivers of patients with stroke sequelae represents a substantial and clinically relevant dimension of post-stroke care in resource-constrained settings. Rather than reflecting only individual or psychosocial factors, the findings suggest that caregiver burden may also be influenced by broader contextual conditions, including the organization of long-term care and the availability of support systems. In this context, caregiver well-being should be considered an integral component of stroke care, rather than a secondary or indirect outcome.

From a health systems perspective, these results support the need to incorporate caregiver-centered approaches into routine clinical practice, particularly in primary care settings. Strategies aimed at improving caregiver preparedness, providing structured support, and facilitating access to community-based resources may help mitigate the impact of informal caregiving. Further research is warranted to evaluate interventions tailored to similar contexts and to better understand how healthcare system characteristics influence caregiver outcomes over time.
